# Functional Morphology and Sexual Dimorphism of Mouthparts of the Short-Faced Scorpionfly *Panorpodes kuandianensis* (Mecoptera: Panorpodidae)

**DOI:** 10.1371/journal.pone.0060351

**Published:** 2013-03-22

**Authors:** Na Ma, Jing Huang, Baozhen Hua

**Affiliations:** 1 State Key Laboratory of Crop Stress Biology for Arid Areas, College of Life Sciences, Northwest A&F University, Yangling, Shaanxi, China; 2 Key Laboratory of Plant Protection Resources and Pest Management of the Education Ministry, Northwest A&F University, Yangling, Shaanxi, China; Sars International Centre for Marine Molecular Biology, Norway

## Abstract

Mouthparts are closely associated with the feeding behavior and feeding habits of insects. The features of mouthparts frequently provide important traits for evolutionary biologists and systematists. The short-faced scorpionflies (Panorpodidae) are distinctly different from other families of Mecoptera by their extremely short rostrum. However, their feeding habits are largely unknown so far. In this study, the mouthpart morphology of *Panorpodes kuandianensis* Zhong et al., 2011 was investigated using scanning electron microscopy and histological techniques. The mandibulate mouthparts are situated at the tip of the short rostrum. The clypeus and labrum are short and lack distinct demarcation between them. The epipharynx is furnished with sublateral and median sensilla patches. The blade-shaped mandibles are sclerotized and symmetrical, bearing apical teeth and serrate inner margins. The maxilla and labium retain the structures of the typical pattern of biting insects. The hirsute galea, triangular pyramid-shaped lacinia, and labial palps are described in detail at ultrastructural level for the first time. Abundant sensilla are distributed on the surface of maxillary and labial palps. The sexual dimorphism of mouthparts is found in *Panorpodes* for the first time, mainly exhibiting on the emargination of the labrum and apical teeth of mandibles. Based on the features of mouthparts, the potential feeding strategy and feeding mechanism are briefly discussed in *Panorpodes*.

## Introduction

Mouthparts are closely associated with the feeding behavior and feeding habits of insects. They have evolved into tremendous diversity in forms and functions following the surrounding change, especially the occurrence of new food sources [1], [2]. As highly integrated structural units, insect mouthparts form direct interfaces to the environment and can be a morphological expression of the feeding strategy of a particular insect taxon [3], [4]. Their features contribute to a better understanding of mouthpart adaptation to various food sources and provide extremely important traits for evolutionary biologists [3], [5], [6]. It is generally accepted that the mouthparts are composed of the same set of homologous components as those derived from a primitive leg-like appendage [7]–[9]. The diverse modifications of the mouthpart morphology can provide valuable data to infer the phylogeny of Endopterygota for systematists [10], [11].

Mecoptera is a small order in Endopterygota with approximately 650 described extant species assigned to nine families [12], [13]. They have been of interest to entomologists out of proportion to their small numbers because of the controversial relative position within Antliophora [14]–[17]. The most remarkable characteristic of Mecoptera is the mandibulate mouthparts located at the terminal end of the long rostrum, which is formed by the prolongation of the subgenae and clypeus [16], [18]. According to previous investigations, the feeding strategies of mecopteran families are quite diverse: Panorpidae and Apterpanorpidae are saprophagous, Bittacidae are predacious, while Boreidae are phytophagous/saprophagous [16], [19], [20].

Panorpodidae is a species-poor family in Mecoptera and only found in the Pacific Rim [21], [22]. This family can be readily distinguished from other mecopterans by the very short rostrum, thus are commonly called short-faced scorpionflies [23]. They currently consist of only two genera, *Brachypanorpa* Carpenter, 1931 occurring in the United States [24], [25] and *Panorpodes* MacLachlan, 1875 distributed in Japan, Korea, China, and the USA [21], [26]–[28]. In *Brachypanorpa*, the rostrum is only slightly longer than the height of the eye, much shorter than that of *Panorpodes*, which is characterized by the rostrum about twice as long as the height of the eye [21], [23]. Based on a successful laboratory work and field observation, *Brachypanorpa* adults are considered to be wholly phytophagous, feeding on small, soft leaves of a wide variety of plant species [24], [25], [29]. However, little is known on the diet and feeding behavior of *Panorpodes*. Although *Panorpodes* is suspected to be phytophagous [26], almost no any direct evidence has been found.

Morphological study of mouthparts can reveal the uncovered feeding habits of insects [11]. Although some previous studies have been conducted on the mouthpart morphology in Panorpodidae, a detailed description of the mouthparts and the functional morphology of this family, especially at ultrastructural level, are still lacking [18], [30], [31].

The objective of this study was to investigate the fine structures of each component of the mouthparts in *Panorpodes kuandianensis* Zhong et al., 2011 at histological and ultrastructural levels, in attempt to partially uncover its feeding habits and explore the role of the each component of the mouthparts in feeding behavior. A profound study of mouthpart morphology may also provide valuable information for systematic and phylogenetic analyses of Panorpodidae.

## Materials and Methods

Adult specimens of the short-faced scorpionfly *Panorpodes kuandianensis* Zhong et al., 2011 were collected from the Huaboshan Forest Park (41°06′N, 125°00′E, elev. 565 m), Kuandian County, Liaoning Province in the northeastern part of China in early July 2012.

For histological observation, the live insects were fixed in Bouin's solution (saturated picric acid: formaldehyde: glacial acetic acid  = 15∶5∶1, v/v) for 24 h before being stored in 75% ethanol. The head was cut down and dehydrated through a series of acetone (from 80% to 100%), infiltrated in series mixtures of Epon and acetone, and finally embedded in Epon 812. After completely polymerized, the samples were cut into semi-thin sections with a Leica EM UC7 microtome, stained with 0.5% toluidine blue, and examined under a Nikon Eclipse 80i light microscope (Nikon Corporation, Tokyo, Japan). Photographs were taken with a CCD digital camera attached to the microscope.

For scanning electron microscopy (SEM), the live specimens were anesthetized in ethyl ether and decapitated quickly. The mouthparts combined with the heads were fixed in 2.5% glutaraldehyde in phosphate-buffered saline (PBS, 0.1 mol/L, pH 7.2) for 12 h at 4°C. The samples were rinsed in the PBS several times and dissected under a Nikon SMZ1500 Stereoscopic Zoom Microscope to acquire all components of the mouthparts. Subsequently, they were dehydrated in a graded ethanol series after ultrasonic cleaning for 30 seconds. Then the samples were dried with carbon dioxide in a critical-point dryer, sputter-coated with gold, and examined in a Hitachi S-3400N scanning electron microscope (Hitachi, Tokyo, Japan) at 15 kV.

### Ethics Statement

No specific permits were required for the described field studies: a) no specific permissions were required for these locations/activities; b) locations were not privately-owned or protected; and c) the field studies did not involve endangered or protected species.

## Results

### Gross Morphology of the Mouthparts

**Figure 1:**
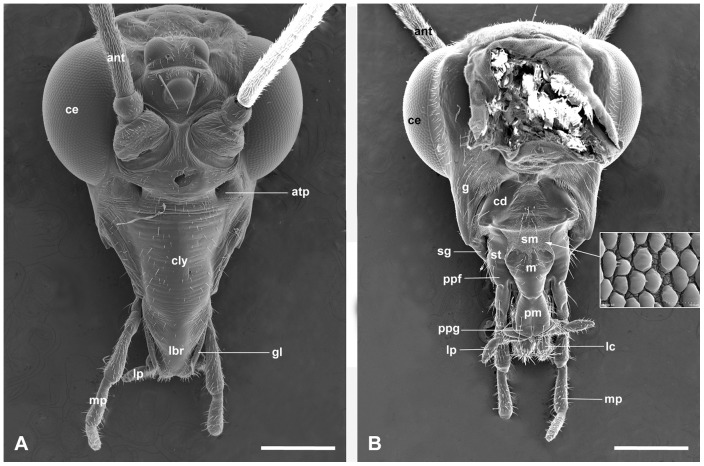
Mouthpart habitus of the male. (A) Anterior view. (B) Posterior view. Inset shows the papilla-like microtrichia on the submentum. ant, antenna; atp, anterior tentorial pit; cd, cardo; ce, compound eye; cly, clypeus; g, gena; gl, galea; lbr, labrum; lc, lacinia; lp, labial palp; m, mentum; mp, maxillary palp; pm, prementum; ppf; palpifer; ppg; palpiger; sg, subgena; sm, submentum; st, stipes. Scale bars  = 500 μm.

The mouthparts of *P. kuandianensis* adults are of typical mandibulate type. The components of the mouthparts, including labrum-epipharynx, mandibles, maxillae, labium and hypopharynx are situated at the tip of a short rostrum, which is formed by elongation of the subrectangular clypeus and subgena (Fig. 1).

**Figure 2:**
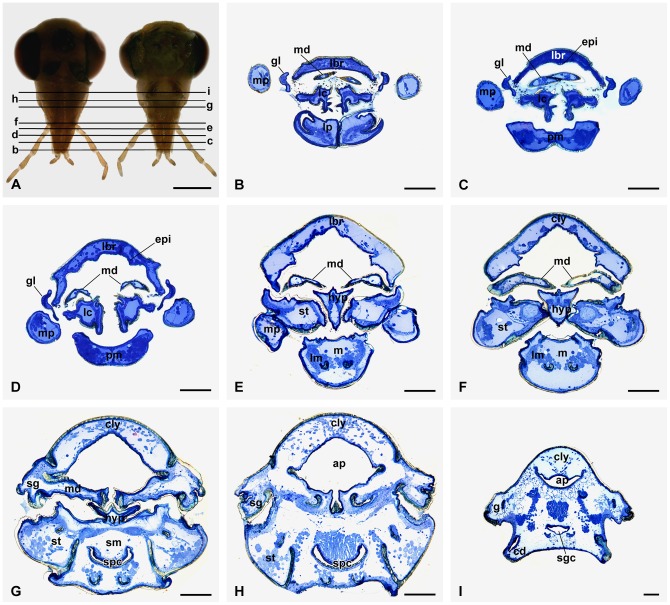
Histological transverse sections of the mouthparts of males. (A) Overview of the sections in both anterior and posterior mouthparts. (B–I) Cross sections of the mouthpart regions for b–i in (A), respectively. ap, anterior pharynx; cd, cardo; cly, clypeus; epi, epipharynx; g, gena; gl, galea; hyp, hypopharynx; lbr, labrum; lc, lacinia; lm, longitudinal muscle; lp, labial palp; m, mentum; md, mandible; mp, maxillary palp; pm, prementum; sg, subgena; sgc, salivary gland canal; sm, submentum; spc, salivary pump chamber; st, stipes. Scale bars: (A)  = 1.00 mm; (B–I)  = 100 μm.

**Figure 3:**
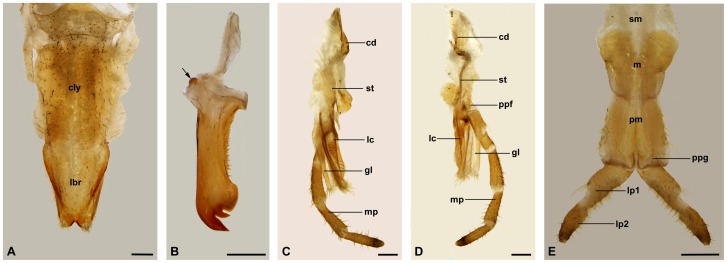
Light micrographs of mouthpart components. (A) Labrum in frontal view, male. (B) Left mandible, female. Arrow shows the condyles. (C) Inner lateral view of right maxillae, male. (D) Posterior lateral view of right maxillae, male; (E) Labium in posterior view, male. cd, cardo; cly, clypeus; gl, galea; lbr, labrum; lc, lacinia; lp1,2, labial palpomere I and II; m, mentum; mp, maxillary palp; pm, prementum; ppf, palpifer; ppg, palpiger; sm, submentum; st, stipes. Scale bars  = 200 μm.

**Figure 4:**
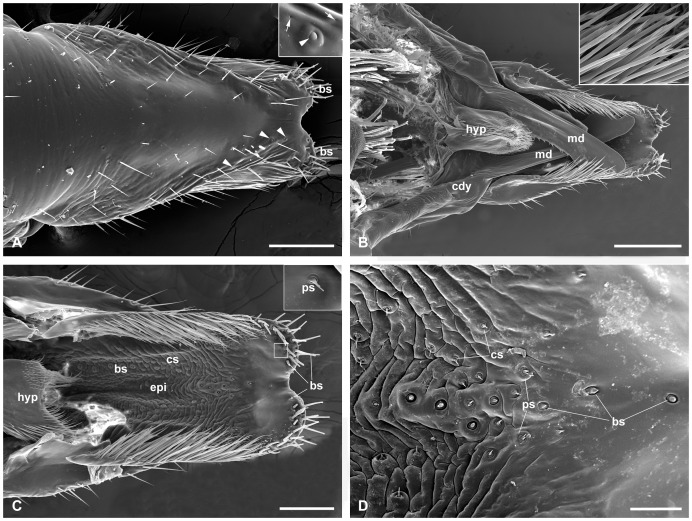
Labrum and epipharynx of male. (A) Anterior view of labium. Inset is the magnification of one of campaniform sensilla (arrowheads) and tiny pores on the surface (arrows). (B) Posterior view of the mouthpart removing maxillae and labium. Inset shows the much-branched microtrichia along lateral edges. (C) Epipharynx. Inset shows the magnification of one palmate sensillum in rectangle. (D) Median sensilla patch of epipharynx. bs, basiconic sensilla; cdy, condylus; cs, chaetic sensilla; epi, epipharynx; hyp, hypopharynx; md, mandible; ps, palmate sensilla. Scale bars: (A) and (B)  = 250 μm; (C)  = 100 μm; (D)  = 25 μm.

**Figure 5:**
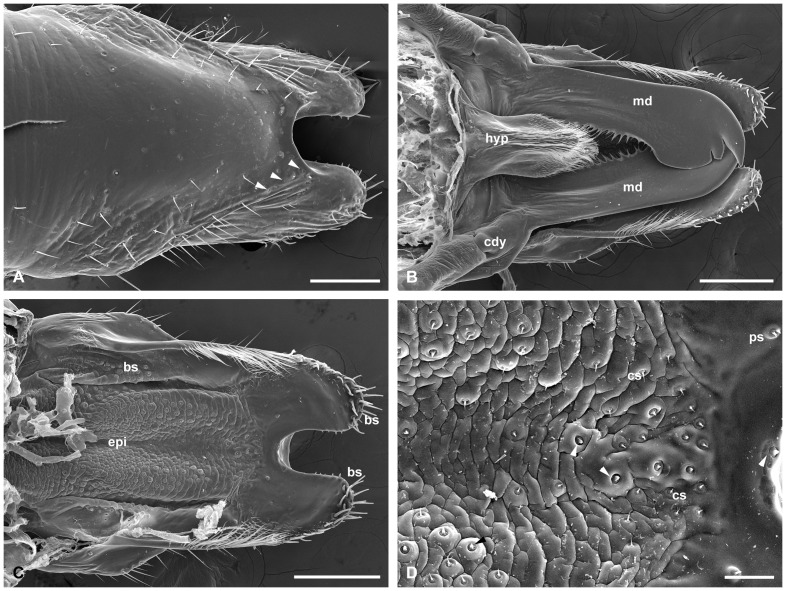
Labrum and epipharynx of female. (A) Anterior view of labium. Note the campaniform sensilla (arrowheads) on the surface. (B) Posterior view of the mouthpart removing maxillae and labium. (C) Epipharynx with sensilla on the membranous surface. (D) Median and apical lateral sensilla patches of epipharynx. Arrowheads show the short basiconic sensilla. bs, basiconic sensilla; cdy, condylus; cs, chaetic sensilla; epi, epipharynx; hyp, hypopharynx; md, mandible; ps, palmate sensilla. Scale bars: (A)  = 150 μm; (B) and (C)  = 200 μm; (D)  = 50 μm.

The frontoclypeal suture is not recognizable, its existence being indicated only by a pair of prominent anterior tentorial pits (Fig. 1A). The setiferous clypeus forms the anterior wall of the rostrum and fused with the labrum distally. The clypeus is semi-circular in cross-section and forms the roof of the food canal (Figs. 2F–I). The clypeolabral suture appears to have vanished, only remaining two deep lateral constrictions (Figs. 1A, 3A). The flattened mandibles are interposed between the labrum-epipharynx and maxillae (Figs. 4B, 5B). The hypopharynx is a small tongue-shaped structure situated in the preoral cavity between the maxillae and the base of mandibles (Figs. 4B, 5B). The paired maxillae are developed, each consisting of a sclerotized cardo and an elongated stipes, an inner lacinia, an outer galea and a five-segmented maxillary palp (Figs. 3C–D). The labium forms the main posterior wall of the rostrum and is composed of the proximal postmentum, distal prementum and paired two-segmented labial palps (Figs. 1B, 3E). Glossae and paraglossae are completely absent in *P. kuandianensis*.

Based on the histological transverse sections of the head, the mouthpart components are separated at the distal one-third and united at the basal one-third of the rostrum (Fig. 2). In the middle region of the rostrum, a preoral cavity is enclosed by the anterior labrum-epipharynx, the lateral maxillae, and the posterior labium (Figs. 2E–G). The hypopharynx is situated in the space between the paired mandibles and maxillae, separating the preoral cavity into the anterior cibarium and the posterior salivarium (Fig. 2F). The orifice of the salivary pump chamber is located at the posterior base of the hypopharynx (Fig. 2G). The anterior pharynx formed by the fused labrum-epipharynx and mandibles occurs almost simultaneously with salivary pump chamber at the submentum center (Fig. 2H). At basal part of the rostrum, the upper anterior pharynx and lower salivary gland canal are visible (Fig. 2I).

### Labrum

The whole labrum is highly chitinized, especially the paired grooves and the apical margin (Fig. 3A). The labrum slightly tapers apically with two lateral sides curving downward (Figs. 3A, 4A, 5A). The lateral sides form a pair of deep grooves in posterior view (Figs. 4B, 5B). The surface of labrum is furnished with some setae, much denser in the two lateral regions and glabrous in the median region. Three or four campaniform sensilla are arranged almost in an oblique line at the apical-lateral regions of the labrum (Figs. 4A, 5A). A few tiny pores are scatteredly distributed on the surface (Fig. 4A). The labrum bears a remarkable emargination at apex (Figs. 4A, 5A). Several basiconic sensilla of varying length are distributed along the edge of apicolateral corners formed by the emargination, with the outer ones longer than the inner ones (Figs. 4A, C). In posterior view, each apicolateral corner is equipped with numerous basiconic sensilla. Among these sensilla, five or six sensilla shorter than 10.0 μm are clustered together near each side of the corner and the remaining setae vary from 20.0 to 50.0 μm (Figs. 4C, 5C). A few palmate sensilla are also found on the inner surface of the apicolateral corners (Fig. 4C). In posterior view, two lateral edges are furnished with dense furcate microtrichia, pointing toward the median line (Figs. 4B–C, 5B–C). The labrum is more even apically and arched basally in transverse section (Figs. 2A–D).

### Epipharynx

The epipharynx is situated on the inner surface of the labrum and basically in accordance with the labrum in shape, except for the paired apical corners (Figs. 4C, 5C). The surface of the epipharynx is membranous and rugose, furnished with many sensilla. Two sublateral broad bands are equipped with abundant basiconic and chaetic sensilla (Fig. 4C). A patch of sensilla is located in the apical median region of the epipharynx. In general, a single basiconic sensillum is located in the top front of the median sensilla patch in both male and female epipharynx (Figs. 4D, 5D). Besides the basiconic and chaetic sensilla, palmate sensilla terminating in a furcate tip are also present in the median region. The epipharynx bears a raised central region in the apical half (Fig. 2C). In the basal half, the epipharynx is gradually concave medially and raised laterally (Fig. 2D).

### Mandibles

**Figure 6:**
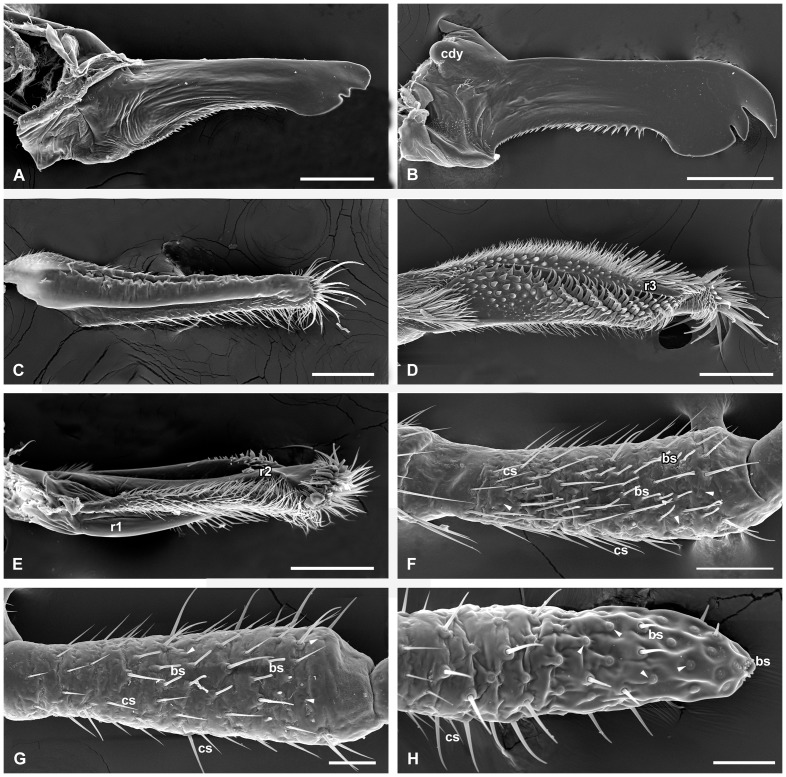
Components of the mouthparts. (A) Anterior view of the left mandible, male. (B) Posterior view of the right mandible, female. (C) Outer-lateral view of the galea, furnished with densely furcate microtrichia laterally and thick spines apically, male. (D) Lacinia in inner posterior view, showing the side between ridges II and III, male. (E) The side between ridges I and II equipped with densely furcated microtrichia, male. (F–H) The third to fifth segments of the maxillary palp, respectively, female. Arrowheads show the campaniform sensilla. bs, basiconic sensilla. cdy, condylus; cs, chaetic sensilla; r1–3, ridge I–III. Scale bars: (A), (D), and (E)  = 150 μm; (B)  = 200 μm; (C) and (F)  = 100 μm; (G) and (H)  = 50 μm.

The left and right mandibles are highly sclerotized and roughly symmetrical, not reaching the distal end of the labrum (Figs. 3B, 4B, 5B). They are articulated with the subgena by anterior and posterior joints (Figs. 4B, 5B). The mandible is blade-like with smooth surface, slightly emarginated and serrated along the middle of the mesal margin (Figs. 6A–B). The apex of the mandible terminates in an expanded lobe, which has two emarginations forming one medial and two lateral teeth (Figs. 6A–B). In dorsal view, the basal region has a slightly rugged concave and setiferous mesal process (Fig. 6A). A middle longitudinal ridge is along its ventral surface until the expanded apical lobe in ventral view (Fig. 6B). The paired mandibles cross to each other near their half length and unite to the subgena at the clypeal base (Figs. 2C, G).

### Maxillae

**Figure 7:**
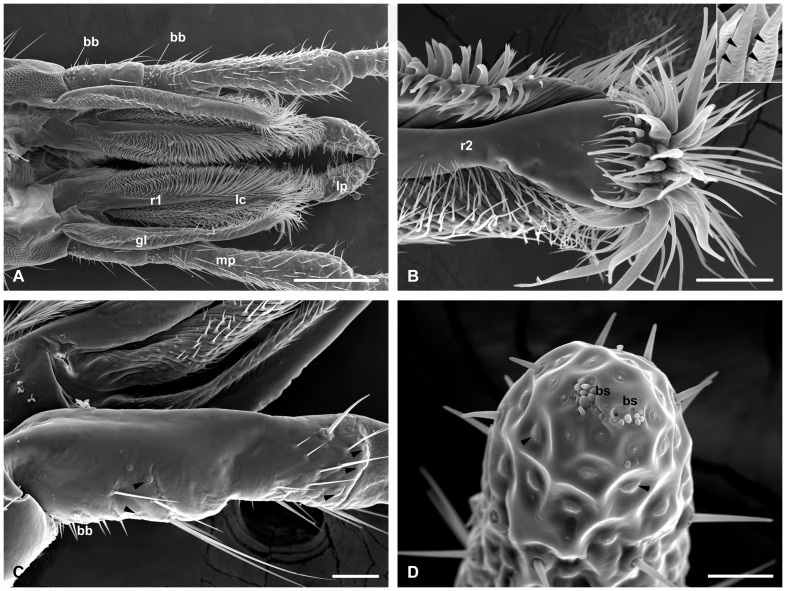
Components of maxilla. (A) Anterior view of the mouthparts with the labrum and mandibles removed, female. (B) Magnification of the thick spines on the maxilla apex. Inset shows the cuticular shafts with several pores (arrowheads), male. (C) The first two segments of the maxillary palp. Arrowheads show campaniform sensilla, male. (D) The distal segment of the maxillary palp, showing the two groups of short basiconic sensilla and campaniform sensilla (arrowheads), male. bb, Böhm's bristles; bs, basiconic sensilla; gl, galea; lc, lacinia; lp, labial palp; mp, maxillary palp; r1–2, ridge I–II. Scale bars: (A)  = 250 μm; (B) and (C)  = 50 μm; (D)  = 25 μm.

The paired maxillae are developed and roughly symmetrical. Each of the maxillae consists of the basal cardo, the middle elongated stipes, the apical galea and lacinia, and the 5-segmented maxillary palp (Figs. 3C–D). The cardo is recognized as a convexity lateral to the base of the submentum, bearing a few setae (Fig. 1B). The elongate stipes is convex along the mesolateral margins and distally connected to the inner lacinia and the outer galea. Several long setae are located along the outer lateral surface (Fig. 1B). The membrane of the lateral cardo and basal stipes connected to the subgena is set with numerous micropapillae (Fig. 1B). The galea is a weakly-chitinized thin lobe (Figs. 3C–D). Its two lateral edges curve inward and form a broad groove facing the lacinia (Fig. 6C). The outer surface of the galea is folded, especially the lateral sides bearing numerous furcate microtrichia. Several stout spines are located on the arc-shaped apex of the galea (Fig. 6C). In natural condition, the paired galeae cover the labrum and lacinia laterally (Figs. 1A, 2B–D). The lacinia is nearly a triangular pyramid, bearing three highly-chitinized ridges that model the lacinia into three sides (Figs. 3C–D, 6D–E). The posterior side between the ridges II and III is slightly concave and furnished with small conical protuberances on basal half and apex-furcate and bended spines in rows on distal half (Fig. 6D). The side with densely furcate microtrichia between ridges I and II faces the groove of the galea (Fig. 6E). The side between ridges I and III is furnished with numerous straight spines, pointing toward the other lacinia (Fig. 7A). The lacinia is furnished apically with abundant long bulgy spines, especially on the expanded apex of the ridge II. The cuticular shafts of these spines bear several pores in lines (Fig. 7B). The lacinia and galea closely contact with each other by the dense microtrichia (Fig. 7A). A bundle of hair brush occurs on the basal median surface of the lacinia (Figs. 6D, 7A). The galea and lacinia fuse together at the base of the labrum (Fig. 2E).

The five-segmented maxillary palp is inserted on a membranous palpifer, which is laterally united with the distal margin of the stipes (Figs. 1A, 3D). The two basal segments are much shorter than others, with the second segment the shortest and about one-third as long as the longest third segment. The fourth and fifth segments are roughly equal in length, and slightly shorter than the third segment (Figs. 3C–D). The surface of the maxillary palp is furnished with numerous sensilla, especially the outer surface. The proximal segment is sparsely covered with several Böhm's bristles basally and long setae apically (Fig. 7A). The inner surface of the proximal segment is almost glabrous, except for a few campaniform sensilla near the setae (Fig. 7C). Besides the similar basal Böhm's bristles, the second segment also bears more dense setae and campaniform sensilla at the apical region (Fig. 7A). The campaniform sensilla are positioned on the apical outer surface and around the apex (Fig. 7C). The remaining three segments are furnished with more sensilla, including the slender chaetic sensilla, blunt-ended basiconic sensilla, and campaniform sensilla that have a raised center surrounded by a depression (Figs. 6F–H). Basiconic sensilla are the main type of sensilla on the third and fourth segments, about 21–24 and 28–30 in number, respectively (Figs. 6F–G). On the fifth segment, the campaniform sensilla are the dominant type of sensilla, at least 50 on its apical half. Two groups of short basiconic sensilla occur on the apex of the distal segment (Fig. 7D).

### Labium

**Figure 8:**
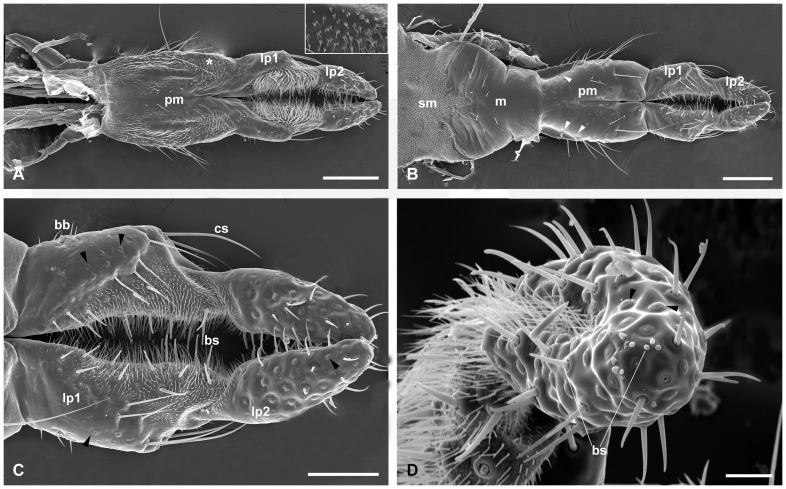
Labium of female. (A) Anterior view of the labium. Inset shows palm-like microtrichia in the asterisk region. (B) Posterior view of the labium. Note the campaniform sensilla (arrowheads) on the lateral side of the prementum. (C) Magnification of the labial palpi. Arrowheads show campaniform sensilla on the two segments. (D) The distal segment of the labial palp, noting the membranous concave and two types of campaniform sensilla (arrowheads). bb, Böhm's bristles; bs, basiconic sensilla; cs, chaetic sensilla; lp1, 2, labial palp I and II; m, mentum; pm, prementum; sm, submentum. Scale bars: (A) and (B)  = 200 μm; (C)  = 100 μm; (D)  = 25 μm.

The postmentum is slightly elongated, occupying the area between the cardines and stipites of the paired maxillae (Fig. 1B). It can be subdivided into the basal membranous submentum and the distal sclerotized mentum (Figs. 1B, 3E, 8B). The proximal half of the submentum is triangular and glabrous except for a few medial setae (Fig. 1B). The submentum is connected to the cardines of maxillae laterally, forming a shallow concavity (Fig. 2I). The distal half of the submentum is concaved mesolaterally and slightly convex mesoposteriorly, furnished with dense, extremely short papilla-like microtrichia (Figs. 1B, 2G–H). The mentum is sclerotized, situated between the apical parts of stipites. It gradually tapers toward apex with a deeply V-shaped concavity mesobasally (Figs. 1B, 8B). The surface of the mentum is glabrous, only sculptured with several transverse furrows on lateral sides and several setae on its basal half (Fig. 8B).

The prementum is separated from the postmentum by a deep fold, medially depressed at the basal two-thirds and cleft apically (Figs. 1B, 3E, 8A–B). The surface of the prementum is highly chitinized posteriorly and bears long setae and campaniform sensilla on the lateral side (Fig. 8B). In anterior view, the surface of the prementum is membranous, covered with numerous unbranched microtrichia basally and palm-like microtrichia distally along the lateral sides (Fig. 8A). The prementum is terminated with a pair of weakly chitinized palpigers and labial palps apically (Fig. 1B).

The labial palps are two-segmented and furnished with numerous sensilla, including Böhm's bristles, basiconic sensilla, campaniform sensilla, and chaetic sensilla (Figs. 8A–C). The proximal segment is broad and longer than the apical segment. The former can be divided into two parts based on the texture, sclerotized basolateral region and membranous mesodistal region. In anterior view, the sclerotized region is glabrous except for a few microtrichia basally and the membranous region is covered with numerous long microtrichia (Fig. 8A). In posterior view, the sclerotized region is furnished with a patch of Böhm's bristles at the base of the lateral side, abundant campaniform sensilla near the apical edge and several long chaetic sensilla at the apex (Figs. 8B–C). The membranous region bears numerous microtrichia, amid with 11–16 long basiconic sensilla (33.0±5.669 μm in length) and several extremely short basiconic sensilla (Figs. 8B–C). Most part of the distal segment is sclerotized, except for the membranous concave mesal surface at the basal two-thirds, which is furnished with 9–11 basiconic sensilla amid in numerous intersecting microtrichia (Fig. 8D). Besides the densely distributed campaniform sensilla, the remaining sclerotized region is also covered with two types of basiconic sensilla, longer ones at the inner sides and shorter ones at the apex (Fig. 8D).

### Hypopharynx

The hypopharynx is a pouch-like structure, raised from the bases of paired mandibles and maxillae (Figs. 4B, 5B, 2H). In anterior view, the hypopharynx upheaves along the median longitudinal line, forming numerous ridges and furrows (Figs. 4B, 5B). Densely furcate microtrichia are distributed along the median line to the distal half of the hypopharynx (Figs. 4B, 5B). From transverse sections, the hypopharynx is obtriangular, situated properly in the space between the mandibles and maxillae (Fig. 2E–F).

### Sexual Dimorphism of the Mouthparts

Although the male and female adults of *P. kuandianensis* bear similar mouthparts, the labrum, epipharynx and mandibles exhibit distinct sexual dimorphism in shape and size.

The labral length is 0.657±0.021 mm (*n* = 12) in males, but 0.783±0.025 mm (*n* = 12) in females. The apical emargination is broadly V-shaped in males and deeply U-shaped in females (Figs. 4A, 5A). Each apicolateral corner of the labrum is equipped with 19–23 basiconic sensilla in males and 22–25 in females. The furcate microtrichia along the two lateral edges of the labrum are much more in males, but relatively less in females (Figs. 4B–C, 5B–C). The difference of the epipharynx between the two sexes lies in that the number of the palmate sensilla in male is more than that of female (Figs. 4D, 5D).

The length of mandible (measured from the articulations to the tip) is 685.7±0.021 μm (*n* = 12) in males but 767.5±0.030 μm (*n* = 12) in females (Figs. 4B, 5B). The whole mandible of the female is much broader than that of the male. Compared with the short serrate teeth directing lateroposteriorly in the male, those of the female are much denser and longer, directing lateranteriorly (Figs. 6A–B). The apical part of the mandible is also much broader in the female. The median and the outer teeth are much longer and more acute in the female than in the male (Figs. 6A–B).

## Discussion

The morphology of mouthparts can reflect the adaptation of insects to various food sources and feeding habits. Because of the important position of Mecoptera in the phylogeny of Endopterygota, the mouthparts have been extensively studied in most families of Mecoptera [10], [18]–[20], [30]–[37]. As far as we know, this may be the first attempt to investigate the mouthparts of Panorpodidae at ultrastructural level.

The mouthparts of the Panorpodidae adults appear to retain a number of primitive traits [18]. Compared with other families, Panorpodidae have the shortest rostrum in Mecoptera [36], [38]. The genus *Panorpodes* bears longer clypeus and labrum compared with the other panorpodid genus *Brachypanorpa* [18], [31]. The epipharynx are equipped with abundant sensilla of gustatory function, including basiconic, chaetic, and palmate sensilla. The lateral and median patches of the sensilla are similar to those of Panorpidae except for the absence of rows of basiconic sensilla apically, but different from the sensillum pattern of *Brachypanorpa* as minute circular areas [31]. Besides the apical teeth that are also present in other families, the mandibles of Panorpodidae are specially characterized by the serrate inner margin [18], [31]. The mandibles of *P. kuandianensis* are similar to those of *P. paradoxa*, but much narrower and longer than those of *Brachypanorpa* [18], [31]. The triangular pyramid-shaped lacinia with dense different spines is observed at ultrastructural level for the first time in *Panorpodes*, with its structure quite similar to that of *Sinopanorpa* [19]. The membranous region of the submentum of *P. kuandianensis* is smaller than that of *Brachypanorpa* and furnished with short papilla-like microtrichia, different from the glabrous membranous region of Panorpidae. It is suggested that the micropapillae are an initial nonspecialized type of membrane armature, underlying which evolve a group of microtrichia and their fusion into the microplates [39].

Although Panorpidae and Panorpodidae are generally considered as sister groups, their feeding strategies are quite different [20], [29]. The adults of Panorpidae are saprophagous, mainly feeding on dead or dying insects and other soft-bodied invertebrates [16]. Before sucking the body fluid of prey, they inject black liquid containing digestive enzymes for extra-intestinal digestion [19], [38], [40], [41]. *Brachypanorpa* is thought to be phytophagous, scraping epidermis from soft leaves of a wide variety of plant species [16]. However, little information has been known on the diet and feeding behavior of *Panorpodes*, except for the finding from some failed rearing trials that they have no tendency to consume insects or other animal material [28]. The adults of *P. kuandianensis* usually stay on leaf surface of the Rosaceae and other herbaceous plants in the understory, leaving on the leaves many small holes that fit the rostrum size of *Panorpodes*. In the collecting season we did not observe abundant flowers that can supply potential floral food for *P. kuandianensis*. In rearing experiments, decaying insects, Rosaceae, and herbaceous plants were provided for *P. kuandianensis* adults as potential food. However, the adults were not observed to consume any of these food items. Only Rosaceae flowers were once accepted by a mating female during copulation (L Jiang, unpublished data). Based on the investigation of the proventriculus of *P. kuandianensis*, some unidentified oblong spores (7.49±2.06 × 2.42±0.30 μm, *n* = 16) were observed to be scattered between the acanthae apart from masses of slurry material in both the males and females (C Yue, unpublished data). The black liquid containing digestive enzymes for extra-intestinal digestion was not observed in Panorpodidae. It seems that the feeding strategy of *Panorpodes* is similar to that of *Brachypanorpa*.

The feeding mechanism may be inferred from the mouthpart morphology of insects. Based on its structure, the labrum is unlikely to move in *Panorpodes*. When they feed, their maxillary and labial palps stretch out. The mandibles bear sharp apical teeth and exclusive serrate mesal margins, with which *Panorpodes* adults are able to cut the leaves into pieces. With the help of their developed maxillae and labium, the adults can further process the plant tissues. Flower-visiting insects generally bear a long air-tight food tube or suctorial proboscis, which can transport the fluid food to the mouth by adhesion or suction [8]. In *Panorpodes*, the epipharynx, mandibles, and hypopharynx work together to form a relatively air-tight food canal, slightly different from that of Nannochoristidae [42]. The apical furcate spines on the galeae and laciniae may play a role of capillarity and serve to direct the flow of fluid from the plant tissues toward the mouth through the food canal [8], [43]. Similar to those of Panorpidae, interdigitated microtrichia of galeae and laciniae may also play a role in filtrating food particles [19]. At the basal part of the cibarium, plant tissues have been processed into slurry, which may flow into the mouth by the direction of the hydrophilic hairs on the hypopharynx. Based on the biological observation and ultrastructural examination, we suggest that the sap of plant leaves is very likely as the main diet of *Panorpodes* adults. The pollens of plants may also be an alternative choice when adults survive unfavorable or harsh conditions.

Overabundance of synonyms is a vexatious problem in taxonomy, frequently encountered and difficult to avoid in taxonomic practices [44], [45]. Some synonyms may be caused by the neglect or unknown nature of sexual dimorphism, which arises through selection for different resource allocation strategies between the two sexes. In Diptera sexual dimorphism is very common in the head structure, especially the mouthparts in those flies in which the female feeds on blood and the male on nectar [46]. In Mecoptera, conspicuous sexual dimorphism is distinctly exhibited in the wings of Boreidae [16]. No sexual dimorphism has been reported on the mecopteran mouthparts, except the sensillum pattern on the epipharynx of *Sinopanorpa tincta* [19]. The labrum and mandibles of the mouthparts in *P. kuandianensis* exhibit stable differences between the two sexes. The males bear a broad V-shaped emargination on the distal margin of the labrum and blunt apical teeth on the narrow mandibles, while the females possess a deep U-shaped emargination on the labrum and sharp apical teeth on the broad mandibles. Although these differences seem unlikely to cause distinct feeding strategy between the sexes, they can at least provide some systematic information, which has not been noticed previously [28].

Although morphological method is not the only approach in systematics, it is still the dominant and important component of our knowledge of the biodiversity [44]. Apart from molecular and biological approaches, more comparative morphological studies are desperately needed to satisfactorily resolve phylogenetic problems in insects.
